# Efficacy and safety of different options for liver regeneration of future liver remnant in patients with liver malignancies: a systematic review and network meta-analysis

**DOI:** 10.1186/s12957-022-02867-w

**Published:** 2022-12-16

**Authors:** Fengming Yi, Wei Zhang, Long Feng

**Affiliations:** 1 Department of Oncology, Second Affiliated Hospital of Nanchang University, Nanchang, 330006 People’s Republic of China; 2 JiangXi Key Laboratory of Clinical and Translational Cancer Research, Nanchang, 330006 People’s Republic of China; 3 Department of Obstetrics and Gynecology, Second Affiliated Hospital of Nanchang University, Nanchang, 330006 People’s Republic of China

**Keywords:** Associating Liver Partition and Portal vein ligation for Staged hepatectomy, Portal vein embolization, Portal vein ligation, Liver venous deprivation, Two-stage hepatectomy, Future liver remnant, Network meta-analysis

## Abstract

**Background:**

Several treatments induce liver hypertrophy for patients with liver malignancies but insufficient future liver remnant (FLR). Herein, the aim of this study is to compare the efficacy and safety of existing surgical techniques using network meta-analysis (NMA).

**Methods:**

We searched PubMed, Web of Science, and Cochrane Library from databases for abstracts and full-text articles published from database inception through Feb 2022. The primary outcome was the efficacy of different procedures, including standardized FLR (sFLR) increase, time to hepatectomy, resection rate, and R0 resection margin. The secondary outcome was the safety of different treatments, including the rate of Clavien-Dindo≥3a and 90-day mortality.

**Results:**

Twenty-seven studies, including three randomized controlled trials (RCTs), three prospective trials (PTs), and twenty-one retrospective trials (RTs), and a total number of 2075 patients were recruited in this study. NMA demonstrated that the Associating Liver Partition and Portal vein ligation for Staged hepatectomy (ALPPS) had much higher sFLR increase when compared to portal vein embolization (PVE) (55.25%, 95% CI 45.27–65.24%), or liver venous deprivation(LVD) (43.26%, 95% CI 22.05–64.47%), or two-stage hepatectomy (TSH) (30.53%, 95% CI 16.84–44.21%), or portal vein ligation (PVL) (58.42%, 95% CI 37.62–79.23%). ALPPS showed significantly shorter time to hepatectomy when compared to PVE (−32.79d, 95% CI −42.92–22.66), or LVD (−34.02d, 95% CI −47.85–20.20), or TSH (−22.85d, 95% CI −30.97–14.72), or PVL (−43.37d, 95% CI −64.11–22.62); ALPPS was considered as the highest resection rate when compared to TSH (OR=6.09; 95% CI 2.76–13.41), or PVL (OR =3.52; 95% CI 1.16–10.72), or PVE (OR =4.12; 95% CI 2.19–7.77). ALPPS had comparable resection rate with LVD (OR =2.20; 95% CI 0.83–5.86). There was no significant difference between them when considering the R0 marge rate. ALPPS had a higher Clavien-Dindo≥3a complication rate and 90-day mortality compared to other treatments, although there were no significant differences between different procedures.

**Conclusions:**

ALPPS demonstrated a higher regeneration rate, shorter time to hepatectomy, and higher resection rate than PVL, PVE, or TSH. There was no significant difference between them when considering the R0 marge rate. However, ALPPS developed the trend of higher Clavien-Dindo≥3a complication rate and 90-day mortality compared to other treatments.

## Introduction

Surgical resection remains the most critical potentially curative treatment for patients with primary liver cancer or metastatic liver malignancies [1, 2]. However, patients’ selection for hepatectomy is limited as the risk of post-hepatectomy liver failure (PHLF), which is life-threatening with severe morbidity and high mortality [3]. The most important method to prevent PHLF is the evaluation of the minimal safe future liver remnant (FLR), which should be 25–30% of the total functional liver volume (TFLV) in patients with a normal liver in the current consensus [4]. Moreover, the minimal requirement FLR volume should be more than 40% of TFLV for patients with chronic hepatitis or cirrhosis [5].

The liver has a powerful regenerative capacity, enabling it to meet the challenge of hepatectomy. To overcome PHLF, some strategies have been developed to induce liver hypertrophy as insufficient FLR before liver resection. Kinoshita et al. reported the first percutaneous transhepatic portal vein embolization (PVE) to promote liver hypertrophy with minimally invasive in 1986 [6], which has become the gold standard for inducing liver hypertrophy with satisfying safety and efficacy [7]. PVE is commonly performed by the percutaneous transhepatic approach. The procedure of PVE includes access to the portal vein and embolization of target vessels [8]. The hypertrophy of segments two or three could reach 52.4% or 32.2% [9]. However, hypertrophy can be diverse, ranging from 28 to 46% at 4 weeks after PVE, which may cause an insufficient increase of future liver remnants and delay the treatment of tumors [8].

Adam et al. introduced two-stage hepatectomy (TSH) for bi-lobar unresectable colorectal liver metastases (CRLM) in 2000 [10]. The highest number of tumors was resected in the first step, and the remaining tumors were resected after a period of liver regeneration. However, only 16 of 398 (4%) became eligible for TSH in patients with conventionally irresectable colorectal metastasis [10]. Portal vein ligation (PVL) was first introduced as a treatment for unresectable liver cancer. It gradually turned into the first step of TSH for treating bi-lobar liver disease, which required laparotomy [11]. PVE and PVL are the standard first-step of TSH [12, 13]. PVL was performed as an occlusion of the target flux of the portal vein. The principle of PVL was like PVE but with more invasive [14]. However, the time to hepatectomy between PVL and tumor resection was more than 30 days [12], which might cause the progress of liver malignancies. Associating Liver Partition and Portal vein ligation for Staged hepatectomy (ALPPS) was firstly reported in 2011 and presented in a milestone study by Schnitzbauer et al. in 2012 [13]. ALPPS includes classical first stage hepatectomy in that all accessible lesions are resected, associated with the transection line of the second stage and the PVL of the diseased liver that should be resected in the “second stage” [14]. The superiority of this procedure is the second procedure’s high success rate, reaching up to 99% of cases [15]. ALPPS has a short time gap between the two stages, but high mortality limits its application [16]. Guiu et al. first reported seven patients treated with liver venous deprivation (LVD) with safety and high efficacy for liver hypertrophy [17]. LVD is a complete trans-hepatic procedure. Combining simultaneous PVE and hepatic vein embolization (HVE) [18]. It showed a 63.3% FLR volume and a 64.3% FLR function increase after extended LVD on day 21 [17].

As the studies show variable efficacy and safety for different options that induce liver hypertrophy for insufficient FLR, a network meta-analysis (NMA) is essential to compare different treatments according to studies published. Herein, we aim to compare the efficacy and safety of PVE, PVL, TSH, LVD, and ALPPS for liver regeneration of future liver remnants in patients with liver malignancies.

## Methods

This study followed the Preferred Reporting Items for Systematic Review and Meta-Analyses (PRISMA) statement.

### Search strategies and selection criteria

We searched PubMed, Web of Science, and Cochrane Library from the database inception up through Feb 2022 for abstracts and full-text articles published comparing different procedures to induce liver hypertrophy in patients with unsatisfied future liver remnants. Keywords for the data search included portal vein embolization, portal vein ligation, ALPPS, two-stage hepatectomy, liver venous deprivation, and future liver remnant. Consensus-based discussions were taken to resolve the authors’ disagreements (FMY and LF).

Studies including prospective and retrospective trials that compared the efficacy and safety of PVE, PVL, TSH, ALPPS, or LVD were selected. We excluded single-arm studies or other combination studies. We chose the most recent or complete study when duplicate publications or studies published in the same center with patients overlapped.

Two reviewers (FMY and LF) of us assessed independently, i.e., the data from each study were subjected to external assessment. The basic information of studies included the author, publication year, study design, disease distribution of patients recruited, cohort, number of patients, countries or regions, age, FLR volume before treatment, and the FLR/TFLV rate. The primary outcome was the efficacy of different procedures, including standardized FLR increase [(post-FLR-beforeFLR)/beforeFLR], time to hepatectomy, resection rate, and R0 resection margin rate. The secondary outcome was the safety of different treatments, including the rate of Clavien-Dindo≥3a and 90-day mortality. The protocol has been registered in PROSPERO (CRD42022354195).

### Risk of bias and certainty of evidence

Newcastle-Ottawa scale (NOS) was used to evaluate the methodological quality of cohort or case-control studies included, which included the following factors: assessment of studies selection, comparability of cohorts, and assessment of outcome [19]. A score≥7 was defined as a high-quality study. For included RCTs, the Cochrane risk of bias tool was used to evaluate the quality, which included the following domains: random sequence generation, allocation concealment, blinding, incomplete outcome data, and selective outcome reporting [20]. Two authors (WZ and LF) evaluated the studies independently and agreed after discussion.

### Statistical analysis

The statistical analysis was conducted using Stata software (version 16, Stata Corp. LP, College Station, TX, USA). Review Manager 5.3 software (Cochrane Collaboration, Oxford, UK) was used to evaluate the risk bias and transform the data that did not report the mean and variances. The heterogeneity of direct and indirect evidence was according to the inconsistency factor and the value of heterogeneity. The studies in the loop were considered consistent if the 95% confidence interval (CI) of the inconsistency factor included 0. The assessment of heterogeneity was according to the *I*^2^ test, and cutoff values of less than 25%, 25 to 75%, and greater than 75% represented low, moderate, and high heterogeneity, respectively. *P* value was used to evaluate global consistency. Network meta-analyses (NMA) of different treatments were according to a random-effects model. League tables and forest plots were generated for back-transformed network estimates. Odds ratio (OR) and mean difference (MD) with 95% confidence intervals were used to compare different treatment options.

## Results

### Search strategy and study selection

**Fig. 1:**
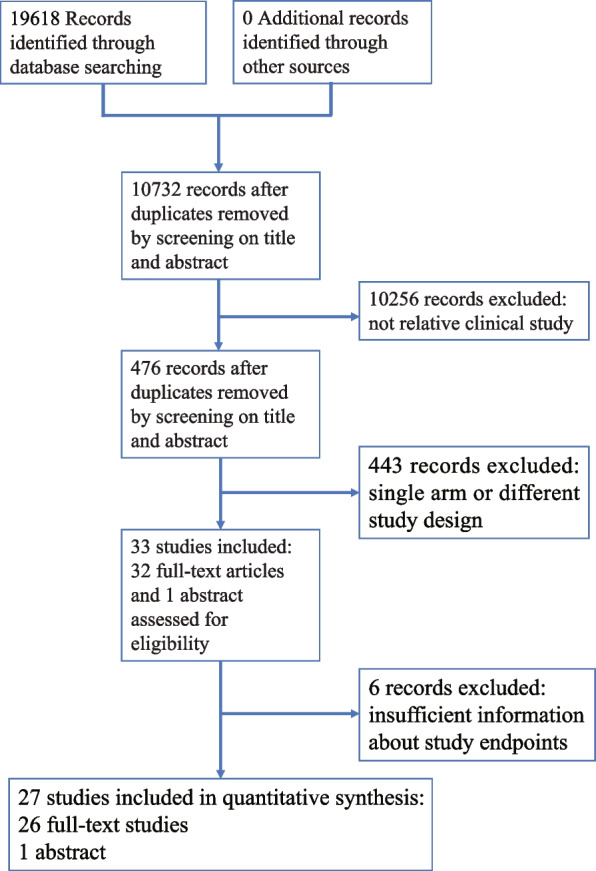
PRISMA flow diagram of screening and selection strategy

A total of 19618 titles and abstracts were identified through database searching; 476 records remained after review of the title or abstracts as duplicates and not relative clinical studies. With a detailed review of the abstract, 33 studies, including 32 full-text articles and one abstract, met the selection standard. Twenty-seven studies were included in quantitative synthesis after removing six records with insufficient information about study endpoints (Fig. 1).

### Baseline characteristics for patients included

**Fig. 2:**
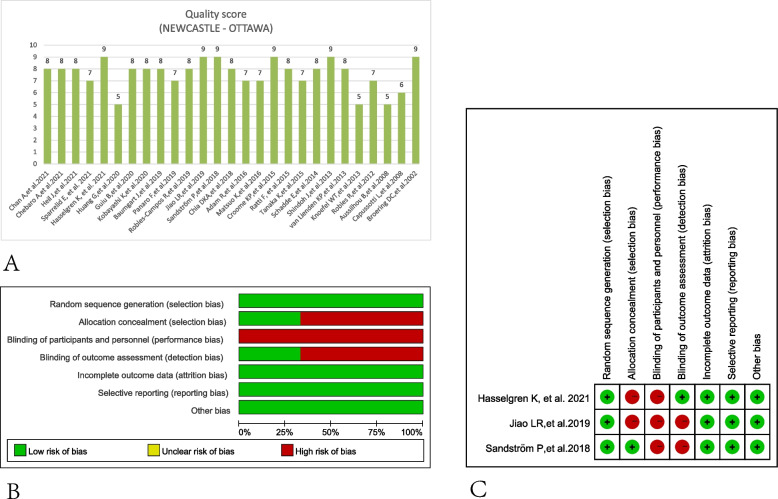
Quality evaluation of studies included. **A** Newcastle-Ottawa scale for cohort or case-control studies. **B** Risk of bias graph for randomized controlled trials. **C** Risk of bias summary for randomized controlled trials

Of the 27 studies recruited, 3 studies were randomized controlled trials (RCTs) [21–23], 3 were prospective trials (PTs) [12, 24, 25], and 21 were retrospective trials (RTs) [18, 26–45] (Table 1). All the participants collected were patients with liver malignancies, including colorectal cancer liver metastasis (CRLM), hepatocellular carcinoma (HCC), cholangiocarcinoma (CCC), neuroendocrine tumor liver metastasis (NETLM), and gallbladder cancer (GBC). The comparative cohort of the studies included ALPPS, PVE, PVL, TSH, and LVD, which compared with each other. As TSH included PVE and/or PVL, they could not differentiate each other in papers compared to TSH with other treatments, so we conducted the conclusions using TSH as original literature. The country distributions of the studies included were mostly European countries, North American, and Asian countries. Age in these trials ranged from 55 to 67 years old, FLR volume before treatments ranged from 200ml to 550ml, and FLR/TFLV ranged from 17 to 33%. Twenty-three of the 27 studies scored ≥ 7 and were high quality (Fig. 2A). The methodological quality of the RCTs was low bias and high quality (Fig. 2B, C).

**Table 1:** Baseline characteristics for patients included

Author/year	Study design	Disease	Cohort	Number of patients	Country/region	Age (years old)	FLR volume before (mL)	FLR/TFLV (%)	Quality score (NEWCASTLE - OTTAWA)
Chan et al. 2021 [26]	RT	HCC	ALPPS	46	China	58.5 (26–80)	302.1 (181.9–524.0)	24.5 (15.7–37.1)	8
		HCC	PVE	102		60 (27–85)	301.1 (142.0–554.0)	24.9 (11.8–44.5)	
Chebaro et al. 2021 [18]	RT	mainly CRLM	ALPPS	85	France	62 (23–82)	348 (95–666)	NR	8
		mainly CRLM	LVD	124		64 (39–81)	379 (161–916)	NR	
Heil et al. 2021 [27]	RT	CRLM/HCC/CCC/GBC/others	LVD	39	Multi-country	63 (2–67)	281 (234–352.1)	18 (16–23)	8
		CRLM/HCC/CCC/GBC/others	PVE	160		67 (58–73)	294 (233–389.7)	18.5 (15–25)	
Sparrelid et al. 2021 [28]	RT	CRLM	ALPPS	71	Scandinavia	65 (56.8–69.3)	NR	21.8 [18.6–25.5]	7
		CRLM	PVE	101		66 (59.4–72.5)	NR	20.9 [17.4–25.3]	
Hasselgren et al. 2021 [21]	RCT	CRLM	ALPPS	48	Scandinavia	64±9	NR	NR	9
		CRLM	TSH	49		63±12	NR	NR	
Huang et al. 2020 [24]	PT	HCC	ALPPS	38	China	NR	NR	NR	5
		HCC	PVE	38		NR	NR	NR	
Guiu et al. 2020 [29]	RT	LM/CCC/HCC/others	LVD	29	France	62 (26–79)	484 (233–805)	22.6 (16.6–37.7)	8
		LM/CCC/HCC/others	PVE	22		66 (45–79)	542 (236–1119)	27.4 (13.7–47.7)	
Kobayashi et al. 2020 [25]	PT	CRLM/HCC/CCC	LVD	20	Switzerland	65 (25–85)	547 (435–656)	35 (28–38)	8
		CRLM/HCC/CCC	PVE	30		65 (41–75)	523 (420–659)	33 (29–40)	
Baumgart et al. 2019 [30]	RT	CRLM	ALPPS	8	Germany	52 (37–69)	NR	NR	8
		CRLM	PVE	14		60.5 (35–74)	NR	NR	
		CRLM	PVL	20		62 (36–78)	NR	NR	
		CRLM	TSH	16		51.5 (42–70)	NR	NR	
Panaro et al. 2019 [31]	RT	HCC/CRLM/others	LVD	13	France	NR	NR	NR	7
		HCC/CRLM/others	PVE	15			NR	NR	
Robles-Campos et al. 2019 [32]	RT	CRLM	TALPPS	21	Spain	66 (44–83)	NR	28 (17–37)	8
		CRLM	TSH	21		59 (47–74)	NR	33 (27–43)	
Jiao et al. 2019 [22]	RCT	CRLM/HCC/others	RALPPS	26	UK	62.4±10.2	NR	23.1±1.2	9
		CRLM/CCC/others	PVE	24		64.3±8.9	NR	23.7±1.1	
Sandström et al. 2018 [23]	RCT	CRLM	ALPPS	48	Norway	65.4 ± 8.9	363 ±85	NR	9
		CRLM	TSH	49		64.9±11.7	365 ± 103	NR	
Chia et al. 2018 [33]	RT	HCC/CRLM/others	ALPPS	10	Singapore	64.7 (51.4–71.1)	337 (202.8–462.5)	21.7 (12.3–28.5)	8
		HCC/CRLM/others	TSH	29		61 (40.6–68.8)	319.5 (209–524.5)	22.2 (15.3–31.9	
Adam et al. 2016 [34]	RT	CRLM	ALPPS	17	France	58 (23–75)	NR	24 (11–38)	7
		CRLM	TSH	41		58 (32–75)	NR	30 (19–53)	
Matsuo et al. 2016 [35]	RT	CRLM	ALPPS	8	Japan	68 (62–78)	303.9±61.1	NR	7
		CRLM/CCC	PVE	14		72 (35–81)	290.2±72.5	NR	
Croome et al. 2015 [36]	RT	CRLM/CCC/HCC/others	ALPPS	15	USA/Canada	55.9±12.1	312.9±84.7	20.1±3.8	9
		CRLM/CCC/HCC/others	PVE	53		59.5±11.3	524.9±219.5	31.4±13.7	
Ratti et al. 2015 [37]	RT	CRLM	ALPPS	12	Italy	59 (51–79)	295±69	22±5	8
		CRLM	TSH	36		59 (42–66)	307±61	23±5	
Tanaka et al. 2015 [38]	RT	CRLM	ALPPS	11	Japan	68 (50–78)	314.2±74.5	NR	7
		NETLM	TSH	54		63 (35–76)	291.4±103.2	NR	
Schadde et al. 2014 [39]	RT	CRLM/HCC/CCC	ALPPS	48	Switzerland	57 (48.5–65)	367 (286–440)	23 (18–29)	8
		CRLM/HCC/CCC	TSH	83		61 (54–69)	389 (324–470)	24 (18–31)	
Shindoh et al. 2013 [40]	RT	CRLM/HCC/NETLM/CCC/GBC/others	ALPPS	25	USA	63 (32–75)	310 (197–444)	NR	9
		CRLM/HCC/NETLM/CCC/GBC/others	PVE	144		58 (33–79)	275 (135–541)	NR	
van Lienden et al. 2013 [41]	RT	CRLM/HCC/CCC	PVL	7	Netherland	59.4±7.6	467 (303–851)	27.7±7	8
		CRLM/HCC/CCC	PVE	14		60.2±11.6	399 (294–517)	25.8±7.5	
Knoefel et al. 2013 [42]	RT	CCC/KT/GC/HCC/CRCLM/NETLM	ALPPS	7	Germany	NR	293±58	NR	5
		CCC/KT/GC/HCC/CRCLM/NETLM	PVE	15		NR	295±94	NR	
Robles et al. 2012 [43]	RT	CRLM	PVL	20	Spain	57 (26–71)	510 (203–824)	NR	7
		CRLM	PVE	18		63 (40–74)	501 (309–703)	NR	
Aussilhou et al. 2008 [44]	RT	CRLM CRNETLM	PVL	17	France	51±14	477±179	NR	5
		CRLM CRNETLM	PVE	18		61±10	509±222	NR	
Capussotti et al. 2008 [45]	RT	CRLM	PVL	17	Italy	63 (52–76)	204 (110–440)	17.7 (9.3–29.5)	6
		CRLM	PVE	31		64 (37–75)	204.5 (125–311)	17.5 (10.7–22.3)	
Broering et al. 2002 [12]	PT	CRLM/HCC/CCC	PVL	17	Germany	63.8±9.2	287.8±60.1	NR	9
		CRLM/HCC/CCC	PVE	17		64.4±6.3	271.8±95.8	NR	

*RCT* randomized controlled trial, *PT* prospective trial, *RT* retrospective trials, *CRLM* colorectal cancer liver metastasis, *HCC* hepatocellular carcinoma, C*CC* cholangiocarcinoma, *NETLM* neuroendocrine tumor liver metastasis, *GBC* gallbladder cancer, *PVE* portal vein embolization, *PVL* portal vein ligation, *TSH* two-stage hepatectomy, A*LPPS* Associating Liver Partition and Portal vein ligation for Staged hepatectomy, *LVD* liver venous deprivation, *FLR* future liver remnant, *TFLV* total functional liver volume, *NR* not report

### Efficacy of different treatments

#### Standardized future liver remnant increase

**Fig. 3:**
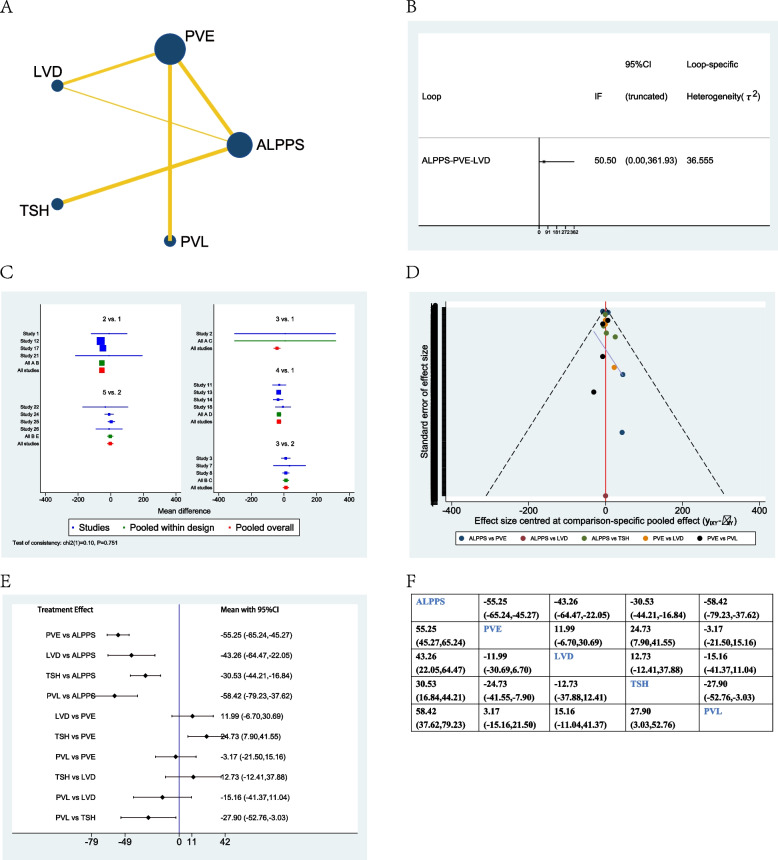
Network meta-analysis of standardized future liver remnant increase. **A** Network plot of studies included, **B** inconsistency test of the loop, **C** global inconsistency between studies, **D** Funnel plot, **E** forest plot of network analysis, and **F** league table of network analysis

Sixteen studies were collected when evaluating standardized future liver remnant increase after different treatment options [18, 22, 23, 25–27, 29, 32, 33, 36, 37, 40, 41, 43–45]. The network plot showed that PVE and ALPPS were the most frequently included techniques in most studies (Fig. 3A). There was no significant inconsistency between the loop ALPPS-PVE-LVD (Fig. 3B). The studies included did not show any global inconsistency (Fig. 3C). The funnel plot demonstrated low publication bias (Fig. 3D).

When considering network meta-analysis of sFLR, the forest plot and league table showed that ALPPS demonstrated the highest regeneration rate when compared to PVE (55.25%, 95% CI 45.27–65.24%), or LVD (43.26%, 95% CI 22.05–64.47%), or TSH (30.53%, 95% CI 16.84–44.21%), or PVL (58.42%, 95% CI 37.62–79.23%). LVD seemed to have a higher regeneration rate when compared to PVE (11.99%, 95% CI −6.70–30.69%) or PVL (15.16%, 95% CI −11.04–41.37%), although there were no significant differences between them (Fig. 3E, F).

### Time to hepatectomy

**Fig. 4:**
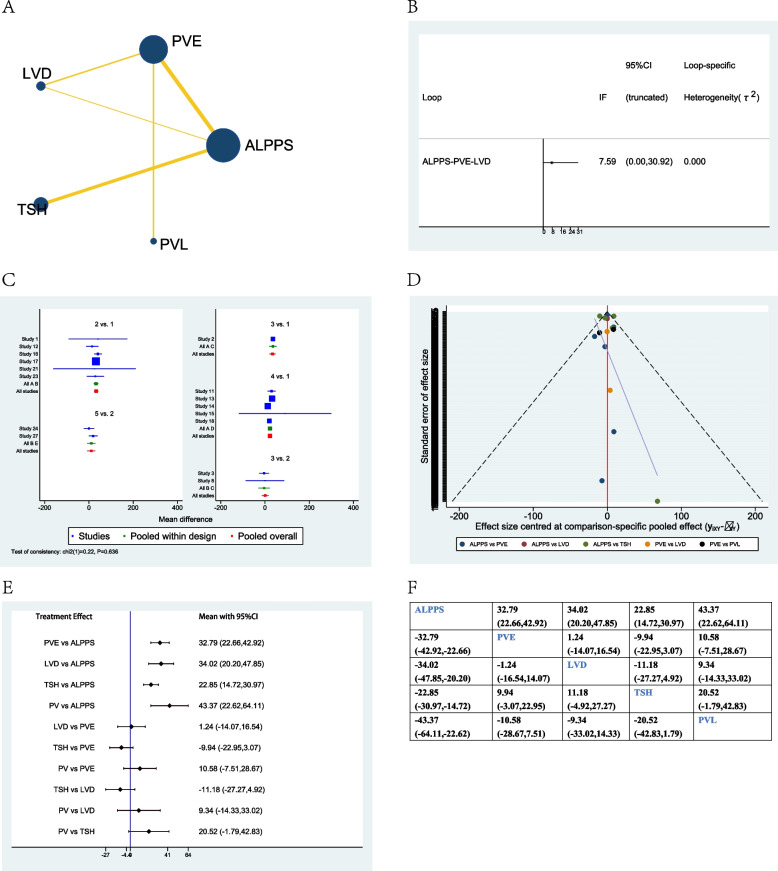
Network meta-analysis of time to hepatectomy. **A** Network plot of studies included **B** inconsistency test of the loop, **C** global inconsistency between studies, **D** funnel plot, **E** forest plot of network analysis, and **F** league table of network analysis

Sixteen studies were included when evaluating the time to hepatectomy between different treatment options [12, 18, 22, 23, 25–27, 32–37, 40, 42, 43]. The network plot showed that PVE and ALPPS were the most frequently included techniques in most studies (Fig. 4A). There was no significant inconsistency between the loop ALPPS-PVE-LVD (Fig. 4B). The studies included did not show any global inconsistency (Fig. 4C). The funnel plot demonstrated low publication bias (Fig. 4D).

When considering network meta-analysis of time to hepatectomy, ALPPS showed a significantly shorter time when compared to PVE (−32.79d, 95% CI −42.92–22.66), or LVD (−34.02d, 95% CI −47.85–20.20), or TSH (−22.85d, 95% CI −30.97–14.72), or PVL (−43.37d, 95% CI −64.11–22.62). However, there were no significant differences between PVE, LVD, TSH, and PVL (Fig. 4E, F).

### Resection rate

**Fig. 5:**
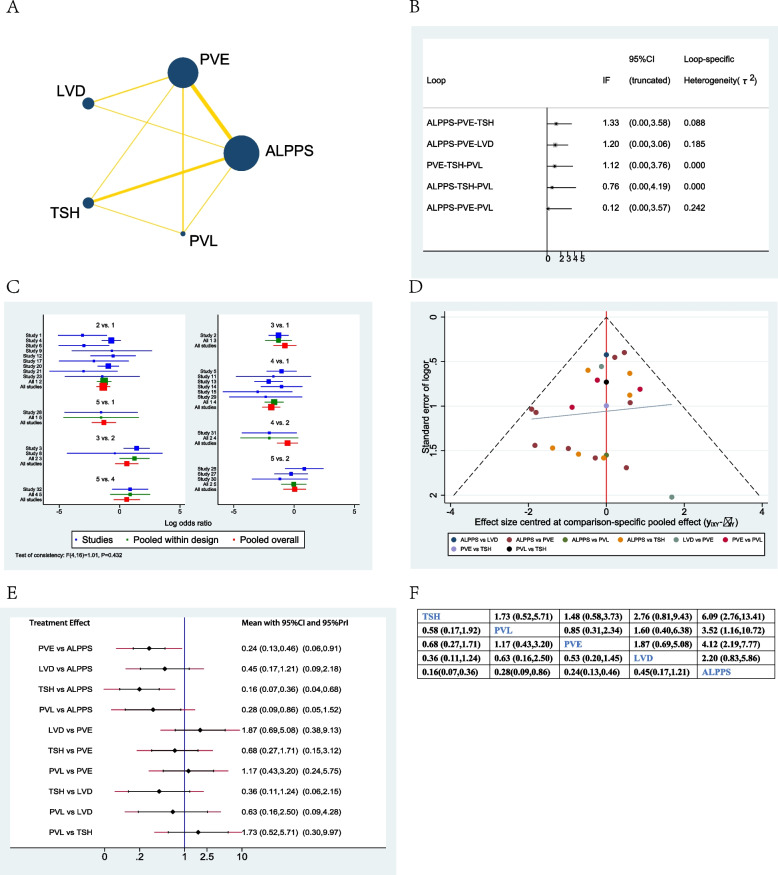
Network meta-analysis of resection rate. **A** Network plot of studies included **B** inconsistency test of the loop, **C** global inconsistency between studies, **D** funnel plot, **E** forest plot of network analysis, and **F** league table of network analysis

Twenty-seven studies were included when evaluating the resection rate between different treatment options [12, 18, 21–45]. The network plot showed that PVE and ALPPS were the most frequently included techniques in most studies (Fig. 5A). There was no significant inconsistency between the loop ALPPS-PVE-TSH, ALPPS-PVE-LVD, PVE-TSH-PVL, ALPPS-TSH-PVL, and ALPPS-PVE-PVL (Fig. 5B). The studies included did not show any global inconsistency (Fig. 5C). The funnel plot demonstrated that the publication bias was not good enough (Fig. 5D), and multiple studies were at the bottom of the funnel.

When considering network meta-analysis of the resection rate of different treatments, ALPPS was considered the highest resection rate when compared to TSH (OR=6.09; 95% CI 2.76–13.41), or PVL (OR =3.52; 95% CI 1.16–10.72), or PVE (OR =4.12; 95% CI 2.19–7.77). Although ALPPS presented a higher resection rate when compared with LVD (OR =2.20; 95% CI 0.83–5.86), the result did not demonstrate a significant difference (Fig. 5E, F).

### R0 resection margin rate

**Fig. 6:**
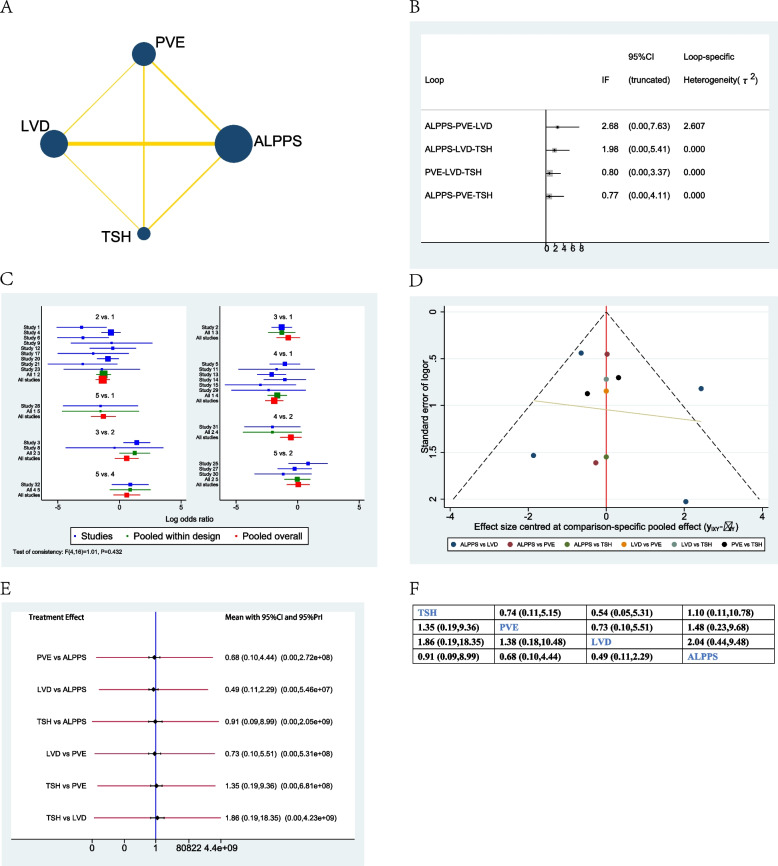
Network meta-analysis of R0 marge rate. **A** Network plot of studies included **B** inconsistency test of the loop, **C** global inconsistency between studies, **D** funnel plot, **E** forest plot of network analysis, and **F** league table of network analysis

Six studies were included when evaluating the R0 marge rate between different procedures [12, 23, 30, 34, 37, 39]. The network plot showed that LVD and ALPPS were the most frequently included techniques in most studies (Fig. 6A). There was no significant inconsistency between the loop ALPPS-PVE-TSH, ALPPS-PVE-LVD, ALPPS-LVD-TSH, and PVE-LVD-TSH (Fig. 6B). The studies included did not show any global inconsistency (Fig. 6C). The funnel plot demonstrated that ted the publication bias was not good enough (Fig. 6D); multiple studies were at the bottom or outside of the funnel.

When considering the network meta-analysis of the R0 marge rate of different treatments, there was no significant difference between ALPPS, PVE, LVD, and TSH in the forest plot and league table (Fig. 6E, F).

### Safety comparison of different treatments

#### Clavien-Dindo≥3a complication rate

**Fig. 7:**
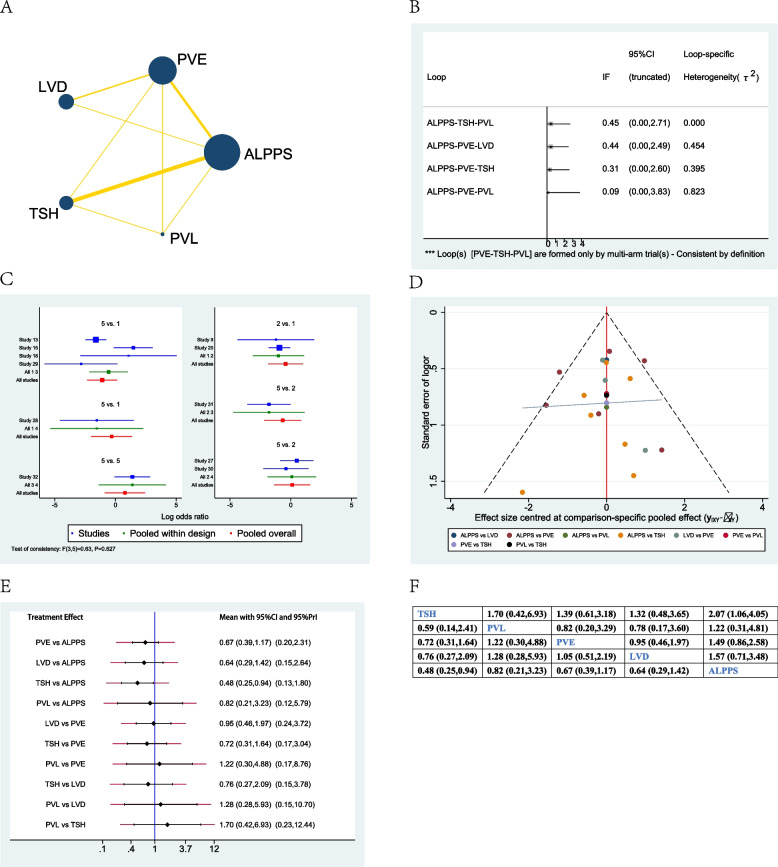
Network meta-analysis of Clavien-Dindo≥3a complication rate. **A** Network plot of studies included **B** inconsistency test of the loop, **C** global inconsistency between studies, **D** funnel plot, **E** forest plot of network analysis, and **F** league table of network analysis

Sixteen studies were included when evaluating clavien-Dindo≥3a complication rate between different options [18, 23–28, 30–35, 37–39]. The network plot showed that PVE and ALPPS were the most frequently included techniques in most studies (Fig. 7A). There was no significant inconsistency between the loop ALPPS-TSH-PVL, ALPPS-PVE-LVD, ALPPS-PVE-TSH, and ALPPS-PVE-PVL (Fig. 7B). The studies included did not show any global inconsistency (Fig. 7C). The funnel plot demonstrated that the publication bias was high (Fig. 7D), as multiple studies were at the bottom of the funnel and the correlation line was not straight enough.

When considering the network meta-analysis of Clavien-Dindo≥3a complication rate of different treatments, there was no significant difference between ALPPS, PVE, LVD, PVL, and TSH in the forest plot and league table. However, ALPPS had the trend of a higher Clavien-Dindo≥3a complication rate compared to other treatments (Fig. 7E, F).

### 90-day mortality

**Fig. 8:**
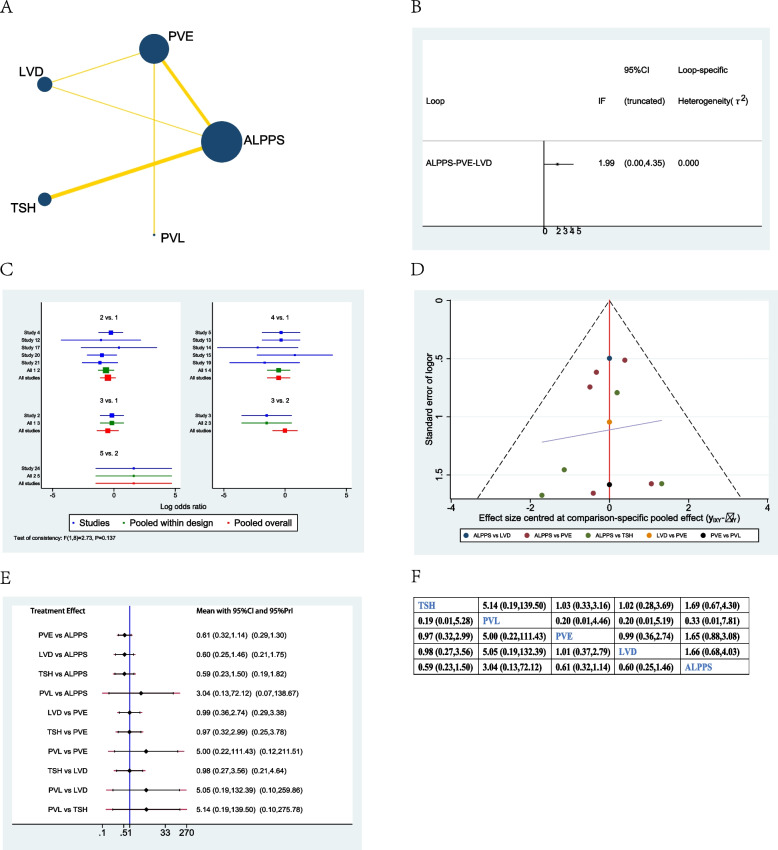
Network meta-analysis of 90-day mortality. **A** Network plot of studies included **B** inconsistency test of the loop, **C** global inconsistency between studies, **D** funnel plot, **E** forest plot of network analysis, and **F** league table of network analysis

Thirteen studies were included when evaluating a 90-day mortality between different treatment options [18, 21–23, 27, 28, 33, 34, 36, 38–40, 43]. The network plot showed that PVE and ALPPS were the most frequently included techniques (Fig. 8A). There was no significant inconsistency between the loop ALPPS-PVE-LVD (Fig. 8B). The studies included did not show any global inconsistency (Fig. 8C). The funnel plot demonstrated that the publication bias was high (Fig. 8D), as multiple studies were at the bottom of the funnel and correlation line was not straight enough.

When considering the network meta-analysis of 90-day mortality of different treatments, there was no significant difference between ALPPS, PVE, LVD, PVL, and TSH in the forest plot and league table; ALPPS and PVL were considered to have the trend of the highest 90-day mortality, although there were no significant differences between all groups (Fig. 8E, F).

## Discussion

This study explores the efficacy and safety of different treatments inducing liver regeneration for patients with insufficient FLR. Standardized future liver remnant increase rate, time to hepatectomy, resection rate, and R0 marge rate were selected to evaluate different options’ efficacy; Clavien-Dindo≥3a complication rate and 90-day mortality were chosen as the evaluation of different treatments’ safety. It is also an updated network meta-analysis for the publication before, which demonstrated just 90-day mortality between ALPPS and other procedures for hepatic hypertrophy [46]. Other publications about the comparison meta-analysis also compare two treatments, PVE and PVL [47], ALPPS, and TSH [48]. Moreover, apart from the FLR volume, the liver function before hepatectomy plays a critical role in predicting PHLF. For instance, ALT and total bilirubin are independent risk factors for PHLF [49], modified albumin-bilirubin grade, Child-Pugh classification, and international normalized ratio are found to be related to PHLF [50].

For the patient’s candidates for partial hepatectomy without enough FLR, the most effective and lowest-risk treatment might be the best option. However, it takes a long time and many effects for the surgeons to explore the most appropriate method for these patients, as if the patients successfully resected the liver malignancies, they would get much more survival time [7]. Although PVL was initially invented to promote liver hypertrophy, it required a surgical procedure with portal pedicle dissection, which could cause high risk and low compliance in candidate patients. PVE has extended time to be set up as the standard procedure for the candidate patients. However, PVE can result in tumor progression in both embolized and non-embolized livers as a long time to hepatectomy [51]. To overcome the defects of PVE or PVL, ALPPS was developed to induce rapid liver hypertrophy to allow liver resection. It included PVL and an in situ splitting of the liver parenchyma, leaving the hepatic artery, bile duct, and hepatic vein intact until the second step of the operation. However, it demonstrated 44% morbidity and 12% mortality, limiting its spread globally [13]. Subsequently, LVD was introduced to defeat the drawbacks; it was a minimally invasive percutaneous procedure that simultaneously abrogated both portal inflow and hepatic venous outflow to accelerate liver hypertrophy [52], which might make a balance between insufficient FLR and tumor progression. However, LVD presented low efficacy on liver hypertrophy and comparable complications [18].

In our study, when considering liver hypertrophy rate, we used standardized future liver remnant increase rate to compare different treatments, as data insufficiency for pure volume increase of the FLR and additional total functional liver volume for each patient. NMA results suggested that ALPPS had the highest regeneration rate compared to PVE; LVD had the trend of higher liver regeneration than PVL or PVE, although there was no significant difference. It was in accordance with the results published before [46, 47]. The reason might be related to no portal vein rerouting in the first stage of ALPPS, which existed in non-ALPPS procedures [14]. When considering the time to hepatectomy, NMA demonstrated ALPPS showed a significantly shorter time when compared to other options. However, there were no significant differences between PVE, LVD, TSH, and PVL. It was in line with the comparison between the ALPPS cohort compared to the PVO cohort [46]. ALPPS reduces the time for the spread of cancer within the short period required for hypertrophy by partitioning the cancer-bearing liver [15]. ALPPS was considered the highest resection rate compared to TSH, PVL, or PVE according to network meta-analysis of the resection rate of different treatments. Although ALPPS presented a higher resection rate when compared with LVD, the result did not demonstrate a significant difference. It indicated that LVD might have a comparable effect on the resection rate compared to ALPPS. There was no significant difference in R0 marge rate between ALPPS, PVE, LVD, and TSH in the forest plot and league. ALPPS ranks as the most promising procedure for liver regeneration from the endpoints discussed. However, safety is also one of the most important factors that we should consider.

We also explored the safety of different treatments but did not find any significant difference between ALPPS, PVE, LVD, PVL, and TSH in the forest plot and league table. However, ALPPS had the trend of higher Clavien-Dindo≥3a complication rate and 90-day mortality compared to other treatments, although there was no significant difference. The results were in keeping with a previous study [46].

Apart from surgical treatment, cell-based therapy has a promising future in promoting liver regeneration. The progress in the bioengineering of stem cells and organoid generation accelerated cell therapy for liver injury [53]. Stem cells have the potential to proliferate and differentiate into substantial mature cells, which indicates the tissue or organ restoration or repairing function in vivo without immune rejection [54]. Stem cells, including induced pluripotent stem cells (iPSCs), mesenchymal stem cells (MSCs), embryonic stem cells (ESCs), endothelial progenitor cells (EPCs), and liver progenitor cells (LPCs) are confirmed to differentiate into hepatocytes or hepatocyte-like cells in preclinical or clinical studies of liver disease [55–57], whereas the efficacy is controversial although the liver function is ameliorated from some stem cells [58]. Numerous protocols are confirmed to generate hepatocytes from iPSCs. iPSCs-derived hepatocytes are promising for the application of disease modeling, drug toxicity testing, and cell transplantation [59]. Hepatic stem/progenitor cells or multipotent stem cell transplantation could lead to donor cell-mediated repopulation of the liver in experimental models of liver injury [56], whereas hepatic progenitor cells (HPCs) are susceptible to malignant transformation by oncogenic mutation cells in an undifferentiated condition within liver microenvironment [60]. Mesenchymal stem cells (MSCs) are used to repair the liver injury and promote liver regeneration. However, the limitations of MSCs administration in liver injury include aberrant differentiation, low engraftment, microvasculature occlusion, and potential tumorigenicity [54, 61]. MSC-based secretome is an alternative cell-free strategy to avoid the potential risk of MSCs, which may contribute to attenuate liver injury and promote hepatocyte regeneration [54].

This network review provides the results of the available evidence for the efficacy and safety of different treatments, but there are still several limitations. Of the studies selected, only 3 were RCTs, 3 were prospective studies, and others were retrospective studies. Therefore, an insufficient sample and selection bias will likely be significant in retrospective and non-randomized prospective studies. Secondly, the impact of different options on recurrence and survival could not be evaluated as a lack of long-term follow-up data. Thirdly, there was inconsistency in the definition of the surgical procedures, which would limit the applicability of these data. Furthermore, the first step of TSH is PVE or PVL; there were no consistent definitions between different studies and could not distinguish the PVE and PVL group in TSH, which might cause selection bias about TSH.

## Conclusion

Our present network study demonstrated that ALPPS has a higher regeneration rate, short time to hepatectomy, and higher resection rate compared to PVL, PVE, or TSH. LVD had the trend of higher liver regeneration than PVL or PVE ranking second to ALPPS and comparable resection rate of ALPPS. There was no significant difference between ALPPS, PVE, LVD, and TSH when considering the R0 marge rate. ALPPS had the trend of higher Clavien-Dindo≥3a complication rate and 90-day mortality compared to other treatments, although there was no significant difference. However, there needs to be more RCTs to verify this evidence.

## Data Availability

The datasets used and/or analyzed in the present study are available from the corresponding author upon reasonable request.

## Abbreviations

FLR: Future liver remnant
NMA: Network meta-analysis
RCTs: Randomized controlled trials
PTs: Prospective trials
RTs: Retrospective trials
ALPPS: Associating Liver Partition and Portal vein ligation for Staged hepatectomy
PVE: Portal vein embolization
LVD: Liver venous deprivation
TSH: Two-stage hepatectomy
PVL: Portal vein ligation
PHLF: Post-hepatectomy liver failure
CRLM: Colorectal cancer liver metastasis
HCC: Hepatocellular carcinoma
CCC: Cholangiocarcinoma
NETLM: Neuroendocrine tumor liver metastasis
GBC: Gallbladder cancers
